# Imperial Typhoid: The Unemployed

**Published:** 1894-01-27

**Authors:** 


### The Hospital

An Institutional Journal of

The Medical Sciences and Hospital Administration, with

Vol. XV.—No. 383.] AN EXTRA NURSING SUPPLEMENT. [Jan. 27, 1894.

### IMPERIAL TYPHOID: THE UNEMPLOYED.

The idea of "defence" is in the air. The nation is just about to spend immense sums of money in defending itself against the possibility of attacks from without. But who is now considering the probability of attacks from within? The unemployed are numerous, more numerous than ever before; and they are strong, stronger than ever before: not strong enough to construct, but quite strong enough to destroy. They are not entirely or even mainly strong because of their numbers, nor yet because of their despair and consequent recklessness: they are strong chiefly because they excite such deep and burning sympathy among the millions of their own order who happen at the moment to be fully employed.

The most urgent business of the moment is defence; and more urgent than defence against outer possibilities is defence against internal dangers. Is there a day passes in which our morning newspapers do not announce to us some strike, some riot, some demonstration of the "unemployed"? The *Times* tells of the doings of this latter class in Liverpool the other day. A large crowd, a rapidly-increasing crowd, assembled in front of a newspaper office to demand, with threats, the name of a person who had written to the newspapers stating that the unemployed would rather starve than work for three-and-sixpence a day. A meeting was held later at the Wellington Monument, at which a man named O'Brien indignantly denounced newspapers and their editors, the town clerk, the city council, and capitalists in general. He saw old ladies, he said, riding in equipages worth at least £500, while they, the workmen, who produced all these fine things, were starving. If he were their head, he vowed, he would lead them into every public building in Liverpool, and show them how to assert themselves. All this is of a piece with what is going on in London, in Yorkshire, in Scotland, and in almost every district and town where large masses of the industrial population are gathered together.

Now, we do not belong to the class of alarmists. On the contrary, we are of those who hold that a large population like our own, consisting of masses of undisciplined, but brave and high-spirited people, can only be governed by a firm hand; and therefore we stand for the very highest degree of firmness which is necessary for the preservation of public order. If shooting be necessary, we are prepared to stand by shooting. But medicine, the teaching medicine of modern times, radical and thorough though it be, never advises the killing of the patient in order to the saving of his life. Our philosophic art approaches even the furious lunatic, who would destroy not only himself but everything and everybody who comes within his reach—it approaches even him with the calmness of science, with the steadfast purpose of goodwill; and so doing, it reduces him to order, and in many instances to happiness and health. This helpful, gentle, but thorough method of modern medicine is the scientific and the rational method which all of us must learn who desire to deal with the internal dangers which threaten the State in the persons of the unemployed and their sympathisers.

Certain of the "physicians" who have charged themselves specially with the sicknesses of the State and with the present outbreak of acute "worklessness," have had long consultations at the Mansion House during the past three months; and they have just issued their diagnosis, accompanied by their prescription; in other words, their report. Reduced to a sentence, their diagnosis is, that the industrial class are suffering from a deficiency of employment, and that, on the whole, things are getting worse: put into a formula their prescription may be written in the two hopeless words, "Non possumus." The patient is getting steadily worse under the old treatment, but unhappily those physicians have nothing but the old treatment to offer.

Is it indubitably certain that the condition of the unemployed is steadily getting worse, and that the unemployed themselves are becoming more threatening as their despair deepens? Mr. Winkler thinks things are getting worse, and he is an authority; Father Lawless thinks they are, and he is an authority; so, too, think Mr. Yarrow and Mr. Vallance, and they are both authorities. All these testimonies are confirmed by the report of the committee itself, assisted by the Charity Organisation Society, and that committee is the greatest authority of all. "The Committee is persuaded," says the report, "that there is an amount of suffering and distress which, if not greatly beyond the average, calls for the most careful and sympathetic consideration of all who can in any way help to mitigate or remove it." Apropos of this, let us see what the committee itself proposes by way of means for mitigation or removal. The sole prescription of the Mansion House Committee, backed by the Charity Organisation Society, is a formal resolution, and the resolution, as we have already stated, contains exactly the ingredients of former prescriptions, and nothing else. Thus runs the resolution: "In view of the existing depression of trade, which has been much aggravated by the coal and other labour troubles, this conference fears that the distress during the coming winter may be severe, and resolves to request the Lord Mayor to urge those of the charitable public who are unable to take part in the duty of relief in the districts with which they are connected, to assist liberally the principal metropolitan charities, which are best able to bestow aid with discrimination and efficiency."

What does this mean? It means that the physicians and specialists have met; that they have talked over the patients' case; that not one of them has anything new to suggest; that they all agree that the case must now be left in the hands of the family practitioner, and that the mixture, of which the sufferer is already weary and sick to death, must, nevertheless, be taken as before. If this be indeed so, all that we can say is that it is a pity. In our judgment the case, if not at the present moment desperate, will rapidly become so. The question is, Can a modern and great nation, which is cram full of money and intellectual capacity, deal successfully with its own residuum, or can it not? We think it can: we are certain it must; or, if it does not, the result will be explosions and destructions of a kind which may be fatal to our national pre-eminence, or even to our national existence. But without threatening one another with huge calamities, are there not hundreds of reasons why we should resolve that we must and will deal effectually with our increasing residuum at all costs? Our failure to deal with it fills us with shame; it covers us with self-contempt. We cannot endure this intolerable frame of mind: we must get rid of it; and there is no other way of getting rid of it but by effectually removing the cause.

There is a new scheme in operation which also has had its Mansion House experiences, though in a strictly private way. That scheme is the "Friendly Workers' Association," already in operation in North Kensington with the most happy and successful results. The merits of this scheme are that it unifies all power, all resources, all aim, and all effort; and that it can be carried out on an imperial scale. Unification is the prime object to be aimed at; and after that universality. Diversity which constantly becomes increasingly diverse, is the destruction of charitable effort. So long as there is central unity, unity of principle, unity of purpose, and unity of plan, the diversity of detail may be not merely harmless, but even useful. But when diversity, which acknowledges no control, is the very centre and principle of all action, how can any result spring from it but confusion, misery, and failure? The "Friendly Workers' Association" aims at uniting Churches and religious organisations, at bringing into service, from the member of Parliament downwards, all capable and willing workers in county, district, parish, or hamlet under one central system and organisation. Where all is darkness, it says, "Let there be light;" where all is confusion it invokes the spirit of order. Is this to be the way out of the darkness? We cannot tell. But at least the leading principle of the Association will be universally admitted to be sound. Let us all unite! That is the Friendly Workers' demand. Let us put away at once mutual opposition and overlapping, and all the failures and miseries they bring in their train. It is quite certain that if all the goodwill, and all the real intellectual capacity which now seek to deliver the poor from the curse of their poverty, will start from one standpoint, and bring themselves into one line of march, all the hopeless poverty of the British nation will fly away at their approach.

